# The contralateral based Cervico-pectoral rotation flap for large neck defects

**DOI:** 10.1007/s10006-021-01022-1

**Published:** 2021-11-21

**Authors:** Naveena A. N. Kumar, Punit Singh Dikhit, Nawaz Usman, Keshava Rajan, Preethi S. Shetty

**Affiliations:** Department of Surgical Oncology, Manipal Comprehensive Cancer Care Centre, Kasturba Medical College, Manipal Academy of Higher Education (MAHE), Manipal, Karnataka 576401 India

**Keywords:** Contralateral cervico-pectoral flap, Large neck defects, Head and neck reconstruction

## Abstract

**Purpose:**

We here describe our technique of contralateral based cervico-pectoral (CCP) flap for the reconstruction of large neck defect following resection of primary tumour or recurrence particularly due to the lymph node mass.

**Methods:**

The study included the patients who underwent major head and neck surgical ablative procedures followed by CCP flap reconstruction between July 2020 and November 2020. Patients were kept on rigorous regular follow-up to evaluate for flap related complications like flap necrosis, flap dehiscence and oro-cutaneous fistula. Among the 5 patients included and presented in the series, 2 patients were salvage cases post adjuvant treatment.

**Results:**

Five patients who have undergone head and neck reconstruction using CCP flap were included. No major flap related complications occurred in post-operative period.

**Conclusion:**

The CCP flap is simple to perform and reproducible and can be added to the armamentarium for the reconstruction of large upper neck defect following resection of primary tumour or recurrence involving the cervical skin in resource limited setting and in contraindication for microvascular reconstruction. Proper planning, meticulous dissection and adequate release or rotation and tension free closure would provide best outcomes.

## Introduction

The reconstruction of the head and neck defects after ablative procedures for primary or recurrent tumour is an age old challenge for surgeon. The reconstructive option should not only provide adequate coverage, but it should also provide a good match for colour, texture and favourable scar location [1, 2]. For the very same reason, number of options available such as local rotation flaps, fasciocutaneous or myocutaneous flaps and the vascularised free flaps. Vascularised flaps although excellent, many a time, are excluded from the armamentarium due to poor skin texture match, atherosclerotic changes in vessels, comorbidities, old age, contraindication for prolonged surgery or due to financial constraints. Thus local flaps are the work horse for the reconstruction in resource limited settings.

When there is a through and through cheek defect along with large outer skin defect involving the neck in cases of oral malignancy, reconstruction becomes more complicated owing to the large defect size, inadequate cover by bi-paddle local flaps, requirement of two local flaps, distance from the donor site and donor site defect closure. It becomes more challenging in recurrent tumours of head and neck, where local flap has already been utilized during primary surgery. The reconstruction of a large neck defect following excision of primary malignancy of neck or following resection of neck skin involved by lymph node recurrence is also demanding due to the necessity of perfect balance between cosmesis and adequate skin cover.

Cervico-pectoral (CP) flap is one of the underutilized flaps which fulfils nearly all the criteria for the defect coverage if planned correctly. The advantages include simple and reproducible technique, use of existing incision only, robust blood supply, good skin colour and texture match and donor area defect [1, 3]. However, ipsilateral cervico-pectoral (ICP) flap may not be possible when large area of donor skin is involved by tumour. In the present study, we have described our technique of contralateral based cervico-pectoral (CCP) flap for the reconstruction of neck defect following resection of primary tumour or recurrence particularly due to the lymph node mass.

## Materials and methods

### Patients

The study was done in the Department of Surgical Oncology of Kasturba Medical College, Manipal. The present case series included the patients, who have undergone head and neck reconstruction using CCP flap between July 2020 and November 2020. A total of 5 patients were identified, details of whom have been discussed in further sections. All flap related complications including flap necrosis, flap dehiscence and donor site complications were documented and managed accordingly.

### Surgical technique

**Fig. 1:**
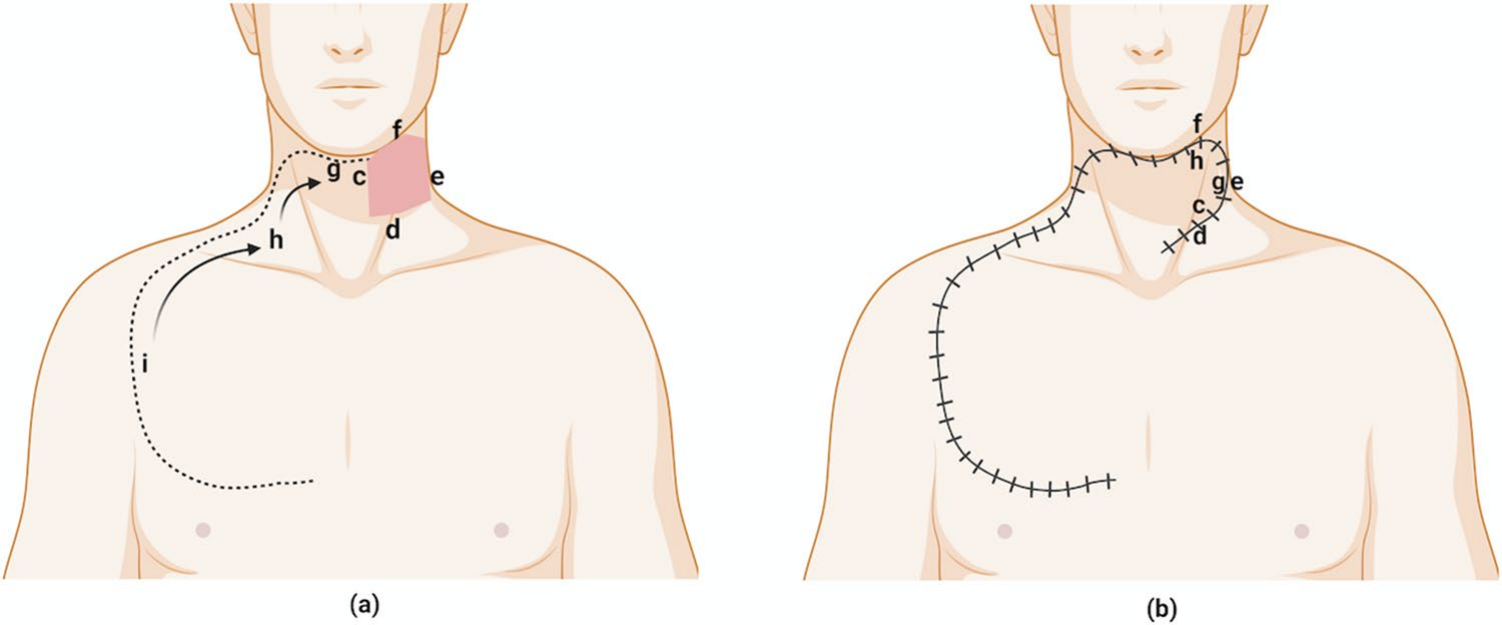
**a** The neck defect (medial wall c, inferior wall d, lateral wall e, superior wall f with CCP flap marking (medial border c, superior border g, lateral border, i.e. cervical part h, pectoral part i). **b** The CCP flap reconstruction. Figure 1 is created with BioRender.com

The neck defect and flap design is marked in the Rose position prior to the commencement of the surgery. The medial wall of the defect is formed by the medial border of the CCP flap (Fig. 1a). The superior border of the flap is marked in the submental and contralateral submandibular area along the inferior border of body of the mandible. Further superolateral, lateral and inferior extension of the flap is planned as is standardly described for an ICP flap [1, 3–5]. We prefer to incise the flap over sternocleidomastoid muscle superolaterally from just below the angle of mandible to the anterior border of trapezius muscle and laterally along the anterior border of trapezius. In case of larger defect, flap can be extended posteriorly behind the ear towards hair line and laterally 2 cm behind the anterior edge of the trapezius [4, 5]. The subsequent extension is over lateral part of clavicle, deltopectoral groove and runs parallel to lateral border of pectoralis muscle [1, 4]. The inferior extension is planned 2 cm above nipple and inferior to third or fourth intercostal space [3, 4]. In case of very big defect, combined CCP and lateral/inferior extension of ICP flap can be planned. If ipsilateral pectoralis major myocutaneous flap (PMMC) is required for inner mucosal reconstruction, its planned in such a way that cutaneous part of the flap is incorporated into the inferior part of the ICP.

The oncological resection is performed with adequate margin. The flap elevation is commenced from the medial and superior border of the flap. The superolateral, lateral and inferior border of the flap can be extended depending on the size of the neck defect. The flap is raised in subplatysmal plane across the midline of the neck and above pectoralis muscle along with pectoral fascia in the chest. Care should be taken to avoid injury to marginal mandibular, greater auricular and spinal accessory nerves. The contralateral neck dissection is performed through same incision if indicated.

The medial border of the CCP flap is sutured to the inferior margin of the defect. The superior border of the CCP flap (submental area and submandibular area) is sutured to lateral wall of the defect. The superior defect is covered by rotation of the contralateral cervical part of the flap. The pectoral part of the flap is mobilized to cover the cervical area (Fig. 1b). After rotation and adequate coverage of the neck defect, contralateral incision is closed primarily.

## Results

Five patients who have undergone head and neck reconstruction using CCP flap were included.

**Fig. 2:**
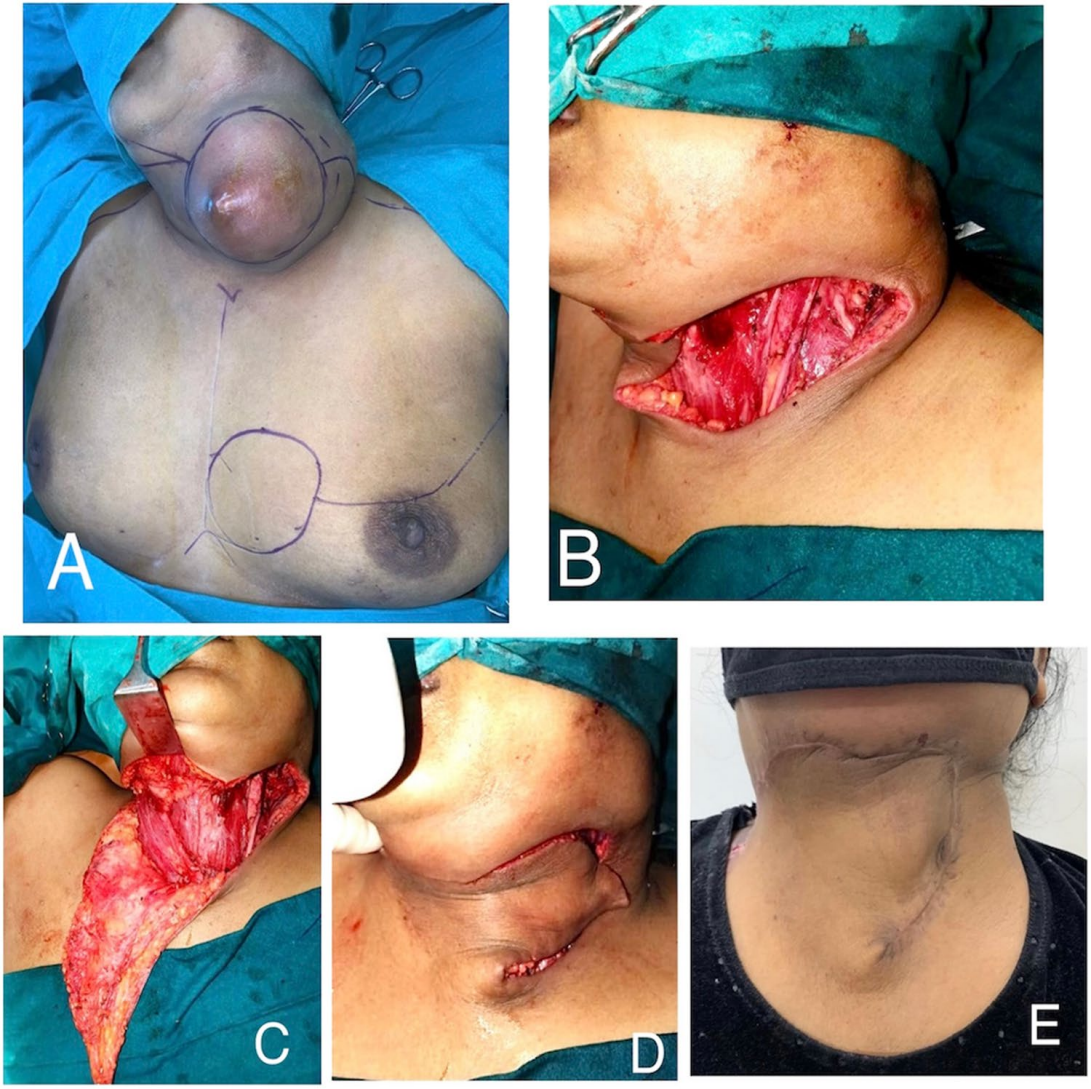
The reconstruction of neck defect using CCP flap following wide excision of primary soft tissue sarcoma of neck

**Fig. 3:**
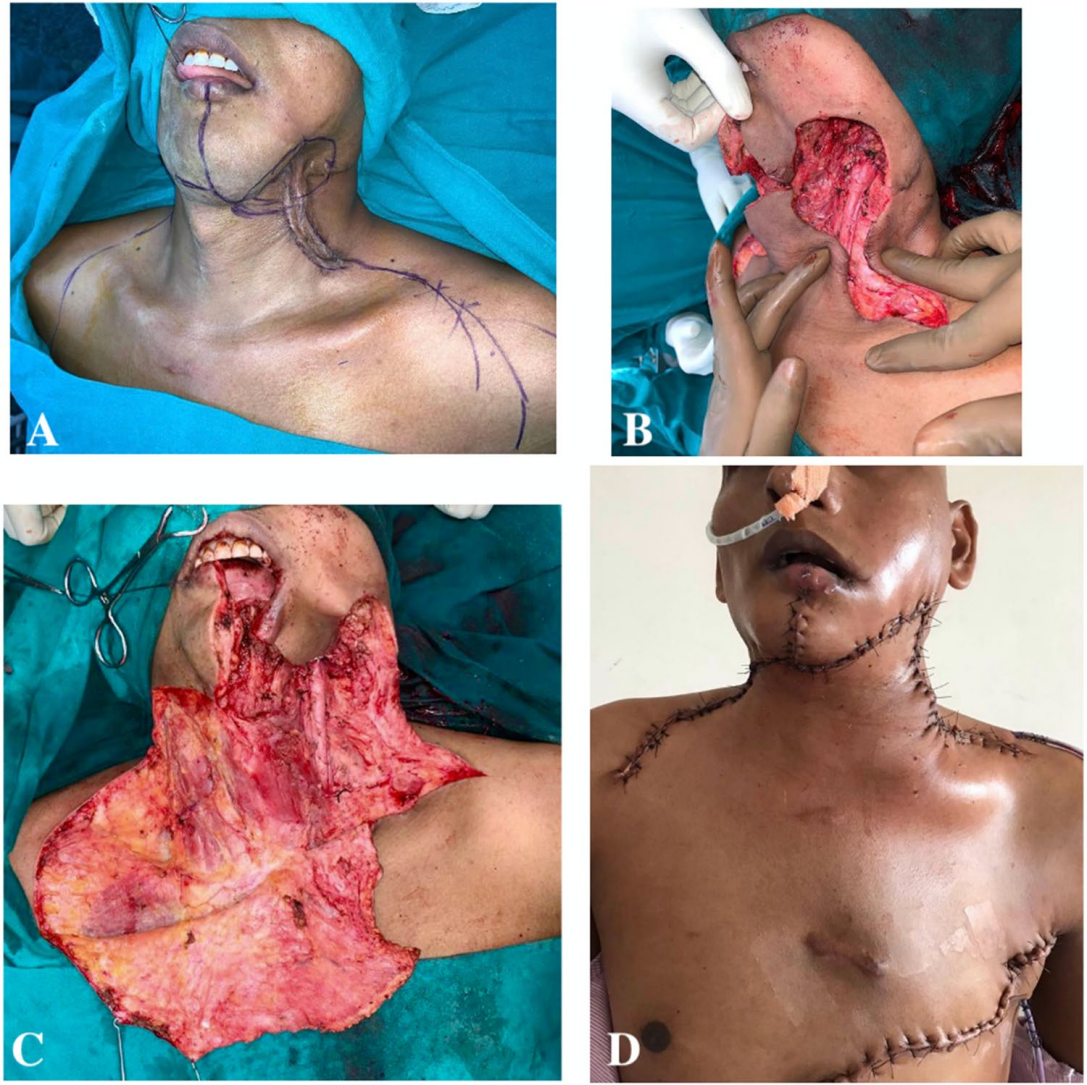
The reconstruction of outer neck skin defect using CCP flap and inner mucosal defect with PMMC flap following composite resection for recurrent cancer of buccal mucosa

**Fig. 4:**
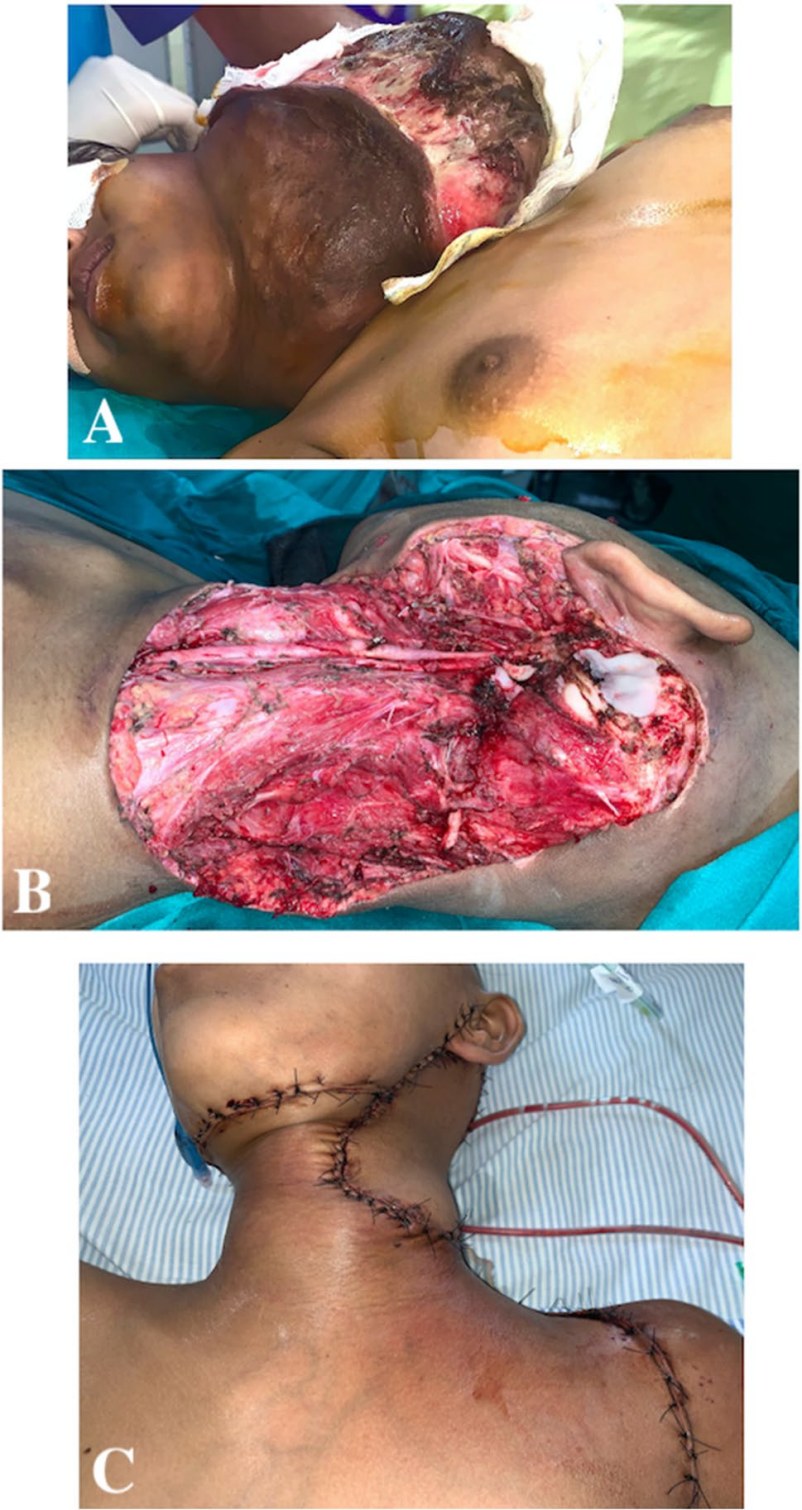
The reconstruction of large neck defect using combined CCP, ICP and latissimus dorsi myocutaneous flaps following wide excision of recurrent sarcoma of neck

1. A 45-year-old lady, diagnosed to have primary soft tissue sarcoma involving the cervical skin. Following wide excision of the tumour, there was a neck skin defect of 10 × 8 cm. A large CCP flap was used with lateral extension up to clavicle. Tension-free closure was achieved with satisfactory post-operative healing. She has completed adjuvant radiotherapy without any flap morbidity (Fig. 2).
2. A 38-year-old male patient known case of oral cavity carcinoma, developed recurrence in left level 1B and II neck lymph node involving skin of cheek and neck. He had previously undergone wide excision, reverse marginal mandibulectomy, modified neck dissection and primary closure and received adjuvant chemo-radiotherapy. Presently, he underwent left segmental mandibulectomy and wide excision of cervical skin, resulting in 9 × 9 cm outer skin defect. A combined CCP flap with a releasing incision up to contralateral deltopectoral groove and ICP flap up to clavicle was used to cover the outer defect, while intraoral defect was closed with pectoralis major myocutaneous flap (PMMC). A 1 × 1 cm area of necrosis at the tip of the CCP was noted in the post-operative period, which was debrided and sutured primarily (Fig. 3).
3. A 15-year-old girl reported to have recurrent sarcoma involving the left anterior and posterior triangle of neck entirely. Wide excision of tumour with ligation of left external carotid artery and left internal jugular vein was performed, resulting in a 15 × 20 cm defect. A combined CCP flap with releasing incision up to contralateral neck, ICP flap and ipsilateral large latissimus dorsi myocutaneous flap were used to cover the defect. Currently patient has completed adjuvant radiotherapy without any flap complications (Fig. 4).
4. A 40-year-old male patient previously underwent wide excision and modified neck dissection for carcinoma lip and received adjuvant radiotherapy was reported to have nodal recurrence in left level 1B involving skin of upper neck. Following wide excision, resultant defect of 8 × 8 cm was covered by combined CCP and ICP flaps.
5. A 70-year-old lady diagnosed to have carcinoma buccal mucosa with large fungating lymph node mass involving right side of upper neck. She underwent right hemi-mandibulectomy, wide excision with modified neck dissection. The resultant 12 × 12 cm outer skin defect was closed with combined CCP and ICP flaps, and intraoral defect was closed by PMMC flap.

All donor sites were closed primarily. No major flap related complications occurred in post-operative period.

## Discussion

The reconstruction following resection of head and neck cancer has come a long way with multiple options including various local and free flaps. Each flap has advantages and disadvantages with respect to extent of cancer, patient and resource factors. In recurrence following multiple cancer-directed treatment and resource limited setting, simple and easily reproducible local flaps are the workhorse of the head and neck reconstruction. We have described the technique of the CCP flap after considering above factors and found it useful in reconstruction of large defects of upper neck.

In 1965 Bakamjian described medially based deltopectoral flap for head and neck reconstruction [6]. Later based on the primary vascular supply, the deltopectoral flap was divided into three zones [7]. The skin from the sternal midline to the deltopectoral groove is supplied by perforating vessels from the internal mammary artery, with the main contribution from the second and third branches. The upper most portion of the deltopectoral flap is predominantly supplied by the thoracoacromial artery. The distal most skin over the deltoid area is supplied by perforators from the deltoid muscle [1, 7]. In 1978, a cervical extension of this flap was described by Becker for reconstruction of large soft tissue facial defects [8]. The skin of the cervical region has rich blood supply from the vessels at the base of the neck and through the platysma [1]. If flap can be designed in a contiguous fasciocutaneous unit, the blood supply from the internal mammary perforators can be preserved along with that of the neck skin forms the basis for the CP flap [1]. Various advantages cited for CP flap are good colour and texture match, easy harvesting and availability of plenty of tissues adjacent to the defect, which can be combined with other locoregional flaps [8–11].

We routinely perform ICP flap for reconstruction of advanced head and neck cancer. The ICP flap may not be feasible in large neck defect due to the involvement of the flap area by the tumour. The good outcomes of ICP flaps have encouraged us to try CCP flap in these patients. With respect to the surgical technique, this flap is simple and easily reproducible. The superior border of the closure line comes underneath the submental and submandibular area, and lateral closure line is quite posterolateral, which helps to avoid obvious scar line. Though our patients show the scar line, we have realized that visibility of the scar line can be masked if flap is planned properly. In our first patient, where only fasciocutaneous flap was required for cover, it can be argued that the free flap may have been ideal. However, CCP flap had advantage of good colour and texture match, easy harvesting and availability of plenty of tissues adjacent to the defect. Only disadvantage is the neck scar, which can be improved with subcuticular sutures. Our technique utilizes contralateral flap and vasculature; thus, it can be utilized in recurrent cases post-surgery and radiotherapy. This is particularly advantageous as compared to ICP flap, as tissue on the ipsilateral side is inadequate and has been exposed prior to the surgical insult and radiotherapy making it less than the ideal option for reconstruction of such large defects. In cases of very large defect, combined bilateral CP flap can be utilized as in our second patient, where most of the defect area was covered by CCP flap. If contralateral neck dissection is indicated, no extra incision is required. Another advantage with our technique is with modification utilizing rotation flap which obviates the requirement for extended release incision as in the first patient. This flap design utilizes same principles as for rotation flap with adequate release. This technique thus provides excellent colour match and aesthetics with minimal scar. The biggest advantage in our technique is its ability to cover cervical defects when used along with other regional flaps like PMMC flap. In contrast when PMMC flap alone is considered for through and through cheek defect, reconstructing a composite defect involving cervical skin is difficult due to limited arc of rotation for bi-paddle PMMC flap in caudal direction. This problem can be addressed by combining PMMC flap for intraoral coverage and CCP flap for extraoral coverage as in second and fifth patient. As in 3rd patient with very large neck defect, CCP flap would help to cover remaining defect after using large latissimus dorsi flap.

The only drawback, which we encountered, was necrosis of flap tip in one patient. This patient had previously received adjuvant chemo-radiation following surgery. Our case series suggest that even after radiation therapy, CCP flap has proven itself to be reliable in covering the defect without compromising or exposing the major vessels of neck on either side. The reason being the robust blood supply of the flap that remained relatively unaffected by the prior therapeutic insults. This flap necrosis was not noted in another patient in our case series who was also received adjuvant radiotherapy following surgery. This problem can thus be prevented by avoiding formation of acute angles during flap raising and adequate release incision with tensionless closure. The operating surgeon should be aware of the fact that releasing incisions form an integral part of the flap raising. Thus, release incisions should be placed in such a way that flap mobilization is not hampered keeping in mind the aesthetic concerns without violating oncological principles. In fact, our case series and ongoing experience suggests that CCP flap is of proven value and benefit in patients with recurrence following surgery, chemotherapy and/or radiotherapy.

## Conclusion

The contralateral based cervico-pectoral (CCP) flap is simple to perform and reproducible and can be added to the armamentarium for the reconstruction of large upper neck defect following resection of primary tumour or recurrence involving the cervical skin in resource limited setting and in contraindication for microvascular reconstruction. Proper planning, meticulous dissection and adequate release or rotation and tension free closure would provide best outcomes.
